# Johnson’s Universal Cyclopedia

**Published:** 1899-08

**Authors:** 


JOHNSON'S UNIVERSAL CYCLOPEDIA.—A new edition, prepared by a corps of thirty-six editors, assisted by eminent European and American specialists, and prepared under the direction of Charles Kendal Adams, LL.D., President of the University of Wisconsin, editor-in-chief. Illustrated with maps and engravings. Complete in eight volumes.

This work, issued from the press in 1898, is revised and brought up full to that date. Its matter has been prepared with great care, and all the subjects in it are brought as nearly as possible to the date of publication. The names of those who prepared the different departments is a sufficient guarantee of the completeness and accuracy of the matter contained. And further evidence of this fact is embraced in the fact that the whole was under the direction of Dr. C. K. Adams, President of Wisconsin State University. This work is one eminently adapted to the needs of the busy man of to-day. Its treatment of the various subjects is sufficiently complete to serve all ordinary purposes, and yet is not tediously prolix. This work should be at the hand of every one who is engaged in or pursues literary work, for ready reference. We can most heartily commend this work to every professional man, and to every student as well. It is published by D. Appleton & Co.

